# Mitochondrial Migraine: Disentangling the angiopathy paradigm in m.3243A>G patients

**DOI:** 10.1002/jmd2.12017

**Published:** 2019-03-14

**Authors:** Jan Smeitink, Saskia Koene, Julien Beyrath, Christiaan Saris, Douglas Turnbull, Mirian Janssen

**Affiliations:** ^1^ Radboud Center for Mitochondrial Medicine at the Department of Pediatrics, Radboud University Medical Center 6500 HB, Nijmegen The Netherlands; ^2^ Khondrion BV Nijmegen The Netherlands; ^3^ Department of Neurology Radboud Center for Mitochondrial Medicine, Donders Institute for Brain, Cognition and Behaviour, Radboud University Medical Center Nijmegen The Netherlands; ^4^ Welcome Centre for Mitochondrial Research, Department of Neurology Newcastle University Newcastle upon Tyne UK

**Keywords:** headache, MELAS, MIDD, migraine, mitochondria, pathology

## Abstract

Migraine, characterized by recurrent attacks of predominantly unilateral throbbing headache, affects approximately 15% of the adult population and is an important cause of disability worldwide. Knowledge required for the development of new classes of antimigraine drugs might come from studying rare metabolic diseases associated with migraine. An illustrative example of a monogenetic disorder associated with migraine is the spectrum of disorders caused by the m.3243A>G mutation in the mitochondrial transfer RNA Leucine. Reported migraine prevalence figures in patients with this particular mutation vary considerably, but compared to the general population, m.3243A>G patients have a higher migraine prevalence. This burdensome symptom might sometimes even be the only clinical feature in maternal relatives carrying the m.3243A>G mutation. Although the exact sequence of events and the relative importance of factors underlying migraine in m.3243A>G MELAS spectrum disorders are still enigmatic, substantial evidence in man exist that dysfunctional mitochondria in both the vascular, the smooth muscle cells and the neuronal system and the interaction between these are at the starting point of the migraine developing pathophysiological cascade. Exclusively based on results of studies performed in patients harboring the m.3243A>G mutation, either in vivo or ex vivo, we here summarize our current understanding of mitochondrial angiopathy associated migraine in m.3243A>G patients which knowledge might lead to potential new avenues for migraine drug development.

## 1 INTRODUCTION

Migraine is a frequently occurring, debilitating form of headache causing a huge socioeconomic burden. Severity ranges from occasional or temporary migraine without aura up to chronic migraine. The underlying pathology of migraine headache is a complex process that demands the recruitment of an intricate set of central nervous system components.^25^ The current view is that activation of the trigeminovascular system, consisting of nociceptive trigeminal sensory afferents surrounding cranial blood vessels, plays an important role in it.^6^, ^7^, ^16^ Pial, dural, and extracranial vessels are part of this system that releases vasoactive neuropeptides from perivascular sensory (pain) nerve fibers and reacts to them with pain sensation (nociception) and vasodilatation.^6^ The fact that all migraine‐provoking molecules are vasoactive and the serotonin 5‐HT receptor agonists like sumatriptan constricts arteries further granted a key role of cranial vasculature in migraine pathophysiology.^79^ Some authors have questioned the vascular hypothesis of migraine and proposed that the cranial arteries response is a negligible epiphenomenon.^6^

The complex pathophysiology of migraine, which is considered to be a polygenetic disease with environmental modifiers, is the main reason for the paucity of migraine‐specific treatments.^37^ Experimental models of migraine in humans, like those that exploit prostaglandin synthesis, have contributed to the discovery of key molecular pathways and the identification of biomarkers and drug targets.^4^ Current paradigms explaining migraine are based on a mixture of results obtained via in vitro experiments in different cellular models, artificial in vivo models and studies in humans, both ex vivo and in vivo.

Here we review the complex relationship between migraine and mitochondria in patients with the spectrum of disorders caused by the m.3243A>G mutation in the tRNA^Leu[UUR]^ an association which has also been disputed.^14^, ^15^, ^23^ Importantly, the results in this review, and the hypothesis presented, are not based on cellular models or migraine in vivo models but are exclusively based on results of studies performed in patients harboring the m.3243A>G mutation, either in vivo or ex vivo.

## 2 MITOCHONDRIA: STRUCTURE, FUNCTION, AND PATHOLOGY

Mitochondria, double‐membrane bound subcellular organelles, are present in every cell of the body except for erythrocytes.^68^ As the brain is a high‐energy consuming organ, brain cells rely heavily on proper mitochondrial functioning. Traditionally, although many more functions are now attributed to it, mitochondria are described as cellular powerhouses.^44^ The OXPHOS consists of five multi‐protein complexes, varying in size from 4 to 44 proteins, and two electron carriers (ubiquinone and cytochrome c). The system is under dual‐genetic control. In the past 20 years, mutations in more than 250 genes affecting OXPHOS functioning have been characterized leading to a great variety in clinical disease expression.^30^ The direct biochemical consequences of these mutations are isolated or combined OXPHOS complex deficiencies causing not only decreased ATP production but also a disturbed redox‐state, increased reactive oxygen species production and abnormal cellular calcium handling to name a few.^43^

During the normal production of ATP, the OXPHOS generates ROS in the form of superoxide through the transfer of excessive electrons to oxygen at specific sites. The most important sites are located in complexes I and III. Mitochondrial ROS represent 90% of total cellular ROS. Under normal conditions highly reactive superoxide is detoxified by the superoxide dismutase enzymes into hydrogen peroxide (H_2_O_2_), a central signaling molecule, which can be further transformed to water by catalase, glutathione peroxidase, or peroxiredoxin enzymes. While ROS play an important role in cell signaling, disturbance in their production level, due to OXPHOS deficiencies, can lead to oxidative stress. Excessive superoxide can react with H_2_O_2_ or nitric oxide (NO), forming respectively the hydroxide radical (OH•) and peroxinitrite (ONOO—). Both reactive radicals can damage macromolecules such as lipids. Oxidative stress is implicated in the pathomechanism of several diseases, including migraine.^28^

## 3 MITOCHONDRIAL DISEASES, MIGRAINE, AND THE M.3243A>G MUTATION

Mitochondrial diseases (MDs) are multi‐system disorders caused by defective oxidative phosphorylation in which the brain, as a high‐energy consuming organ, is frequently affected. Although the brain weight/body weight ratio is low (an adult brain weighing approximately 1.5 kg), the brain consumes 20% of oxygen and 50% of glucose delivered from the vasculature for ATP synthesis.^5^ Neuronal cells rely heavily on OXPHOS while glial cells also contain glycogen as an energy source.^47^ Most energy consumption in the brain is used for synaptic transmission and delivered by mitochondria, preferentially localized to pre‐ and postsynaptic sites.

The range of central nervous system manifestations in MD is broad.^76^ Migraine is a frequently reported symptom, especially in m.3243A>G spectrum disorders. M.3243A>G patients have higher migraine prevalence than the general population.^61^ Migraine is sometimes even be the only clinical feature in maternal relatives carrying the m3243A>G mutation.^19^

Reported migraine prevalence figures in m.3243A>G patients vary considerably. One explanation for these observed differences in reported migraine prevalence figures in m.3243A>G patients is that, based even on the mutation only, a spectrum of disorders has been included in the studies, thereby not discriminating for example, classical MELAS patients with stroke‐like episodes from maternally inherited diabetes and deafness (MIDD) patients, of which the former population is more severely affected and more likely to exhibit migraine. Besides, differences in data gathering via the use or nonuse of specific questionnaires, interviews, and clinical examination might have contributed to the reported differences.^1^, ^22^, ^33^, ^46^, ^51^, ^52^, ^61^, ^83^ In cohort studies using the NMDAS (Newcastle Mitochondrial Disease Score: Section II—System Specific Involvement) questionnaire, migraine was present in ~18%‐29% of the patients.^22^, ^51^, ^62^ These figures are significantly lower than in three recently published large studies in which (adult) patients underwent a structured diagnostic headache interview using an operational diagnostic tool following the International Headache Society criteria.^33^, ^83^ In patients harboring the m.3243A>G mutation (associated with for example, MELAS syndrome) the percentage of migraine ranged between 38% and 85%.^33^ (Table 1).

**Table 1:** Migraine in mitochondrial disease^a^

Mutation	Phenotype	N total	N headache/migraine (%)	Type study	Author year
m.3243A > G	mixed	71	13 (18%)	Prospective registry	De Laat 2012^22^
m.3243A > G	mixed	129	30 (23%)	Retrospective	Nesbitt 2013^61^
m.3242A > G	mixed	133	35 (27.8%)	Retrospective	Mancuso 2014^51^
m.3243A > G	mixed	57	33 (58%)	Questionnaire	Guo 2016^33^
m.3243A > G	MELAS	8	7 (88%)	Questionnaire	Kraya 2017
m.3243A > G	MELAS	13	5 (38%)	Questionnaire	Vollono 2017^83^

The 1 year prevalence of migraine in the general population is 12%. The annual and lifetime prevalence are 18% and 33% in women, respectively, and 6 and 10% in men.^24^

The frequency of migraine headache described in these studies varied from once per month to more than 20 episodes per month. Many patients with migraine and probable migraine show accompanying symptoms such as vomiting, photo‐, phonophobia, or an increase of headache following physical activity. It has been reported that the duration of attacks in patients with mitochondrial migraine was shorter than in patients with non‐mitochondrial migraine.

Potentially raised ROS in neurons and cerebral vasculature due to OXPHOS deficiency will have a major impact, also due to the fact that brain tissue by itself is susceptible to oxidative stress (for reviews see^12^, ^28^). Ex vivo indications of increased ROS or its consequences in m.3243A>G patients have been obtained by studying serum samples of 14 patients with this mutation showing increased mean levels of daicron‐reactive oxygen metabolites (d‐ROMS) and lowered biological antioxidant potential (BAP)/d‐ROMS ratio.^40^ In non‐mitochondrial migraine patients increased oxidative stress markers like malondialdehyde, total antioxidant activity, GSH/GSSG concentrations were observed as were decreased concentrations of plasma GSH, glutathionine‐S‐transferase (GST), and total antioxidant capacity compared to controls.^40^, ^80^

PET imaging with ^56^Cu‐ATSM and ^18^FDG demonstrated increased oxidative stress and increased glucose metabolism following hyperemia in a MELAS syndrome patient with stroke‐like episodes.^41^ Importantly, to our best knowledge, so far none of these imaging studies have been done in migraine m.3243A>G patients without stroke‐like episodes. More advanced ROS detecting imaging probes like MitoNeoD, currently still under investigation in animal models, might contribute to a better understanding of the complex relationship between ROS‐redox metabolism, mitochondrial dysfunction and mitochondrial migraine.^73^

## 4 ANATOMICAL, PATHOLOGICAL, BIOCHEMICAL, CELL BIOLOGICAL, AND ELECTROPHYSIOLOGICAL FINDINGS IN THE BRAINS OF M.3243A>G PATIENTS

Montagna and coworkers were among the first to suggest an association between mitochondrial dysfunction and migraine.^58^ Yorns and Hardison summarized the different types of evidence between mitochondrial dysfunction (biochemical, morphological, genetic, and therapeutic) further supporting an association between mitochondria and migraine.^85^ Since then, numerous others pinpointed to this association (for a recent review see Ferroni et al.^28^).

**Figure 1:**
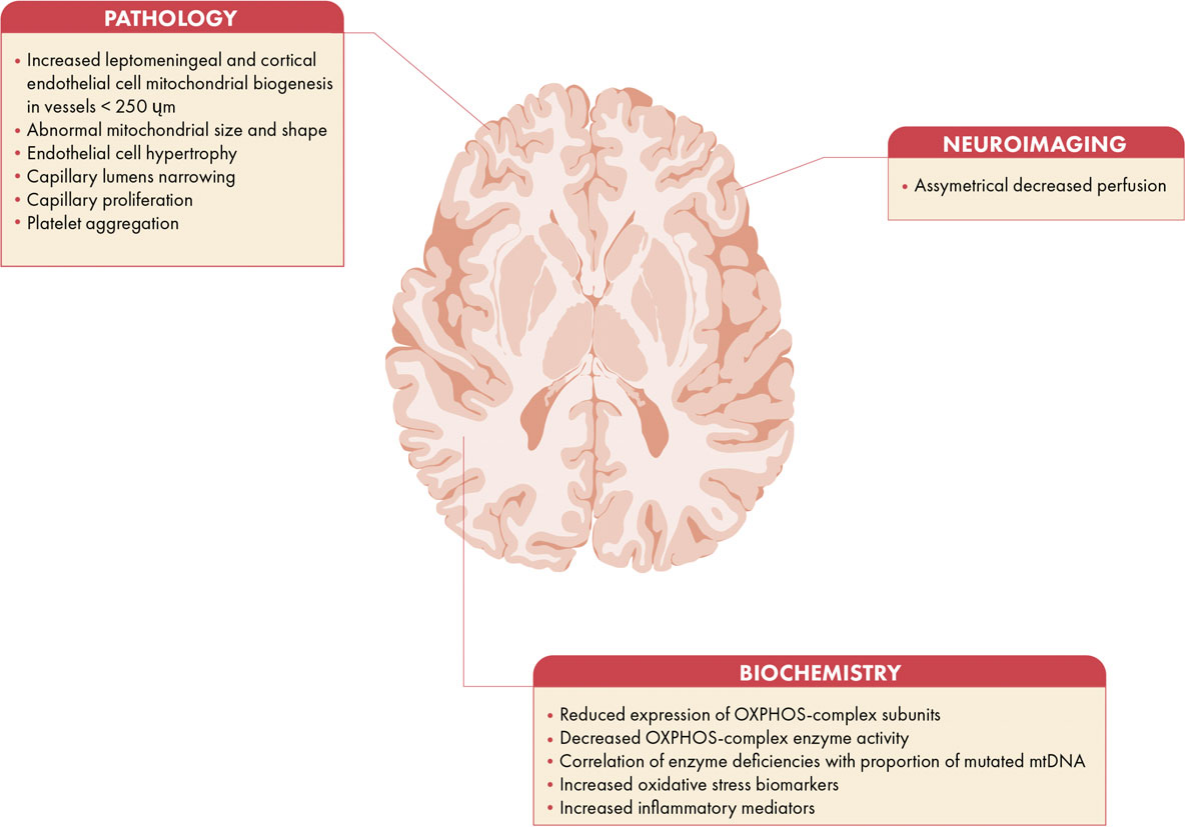
Pathological, neuroimaging, and biochemical features associated with mitochondrial migraine

An important question is what explains the high prevalence of migraine headache in m.3243A>G patients or in other words what is the underlying pathophysiological mechanism? Here, we aim to answer this question based on available data in literature in humans. The reasons to focus on patients with the m.3243A>G mutation is based on, the m.3243A>G mutation prevalence from 7.59 to 236/100.000,^17^, ^49^, ^54^ on the relatively high prevalence of migraine in syndromes caused by the m.3243A>G mutation and on the relatively high number and variety of brain investigations performed in this patient population. Figure 1 summarizes the most important pathological, biochemical and neuro‐imaging abnormalities observed in m.3243A>G patients.

### 4.1 The m.3243A>G mutation causes a spectrum of disorders

The first report mentioning migraine in MELAS patients dates back from 1988.^57^ The acronym MELAS (mitochondrial encephalopathy, lactic acidosis, and stroke‐like episodes) was first described as a separate disease entity more than 30 years ago.^66^ The disease causing mutation, an A‐to‐G transition at nucleotide pair 3243 in the dihydrouridine loop of the mitochondrial tRNA^Leu(UUR)^ was reported 6 years later.^32^

Our current understanding is that the *MT‐TL1* m.3243A > G mutation is associated with a spectrum of diseases involving not only the classical MELAS presentation but also MIDD (maternally inherited diabetes mellitus and deafness)^21^, ^38^, ^53^ with or without hypertrophic cardiomyopathy and renal failure, CPEO^36^, ^59^ and mixed‐phenotypes showing a variety of different complaints, signs and symptoms.^81^ One explanation for the observed variation in phenotypic expression is “heteroplasmy” where wild‐type mitochondrial DNA coexists with mutated molecules in the same cell and different cells have different heteroplasmy percentages.^13^

### 4.2 Mitochondrial angiopathy: a frequent finding at autopsy in MELAS syndrome patients

The pathological features of MELAS syndrome include the accumulation of abnormal mitochondria in endothelial cells and smooth muscle cells of the pial arterioles, on the brain surface and penetrating in the Virchow‐Robin space, and in small intracerebral arteries.^39^ In more contemporary wording “accumulation” refers to an increased number of mitochondria that might be caused by increased biogenesis, a phenomenon sometimes also encountered in skeletal muscle fibers and then termed “ragged red fibers,” or by a decreased mitochondrial autophagy referred to as “mitophagy.”^69^ The accumulated brain vascular endothelial and smooth muscle cell mitochondria may have abnormal sizes and shapes and inclusions, as seen in skeletal muscle fibers of affected patients. Ohama et al.^64^ performed systematically electron microscopy (EM) studies of the cerebral blood vessels in two patients with MELAS (Table 2). They found marked accumulation of mitochondria in the cell bodies of smooth muscle cells and endothelial cells and numerous smooth muscle cells showing degeneration or necrosis in the tunica medica. These abnormalities, which they termed mitochondrial angiopathy, were most prominent in the walls of pial arterioles and small arteries up to 250 μm in diameter, and less frequent and severe in the larger pial arteries and intracerebral arterioles and small arteries. Kishi et al^42^ performed autopsy on a 30‐year‐old woman. EM of the brain capillaries of this patient revealed hypertrophy of the endothelial cells, increased mitochondria in the subendothelial space, and narrowing of the capillary lumens. Mizukami et al. described the autopsy in a 38‐year‐old male^56^ showing that both cerebral and cerebellar blood vessels had marked aggregation of mitochondria in smooth muscle cells and the vascular endothelium. Bertrand et al.^7^ reported numerous focal and so called pseudolaminar cortical necrosis in the brain of a 27‐year‐old MELAS patient, regardless of vascular supply, with characteristic proliferation of capillary vessels. Brain at autopsy of a 47‐year old woman with MELAS syndrome showed vascular sclerosis, cerebral atrophy, diffuse cortical gliosis, basal ganglia calcifications, and a small remote left temporal lobe infarct.^67^ The molecular neuropathology of two patients with the m.3243A > G mutation showed that the most severe COX deficiency associated with the highest proportion of mutated mitochondrial DNA was present in the walls of the leptomeningeal and cortical blood vessels in all brain regions.^8^, ^20^, ^31^ Betts and coworkers concluded that vascular mitochondrial dysfunction is important in the pathogenesis of the stroke‐like episodes in MELAS patients. They suggested, as migraine is a commonly encountered feature in MELAS syndrome, that the vascular mitochondrial dysfunction together with cortical spreading depression (CSD) might underlie the selective distribution of ischemic lesions in the posterior cortex in these patients.^8^ Filosto et al. compared the neuropathology of six different mitochondrial diseases with respect to the distribution and severity of the lesions.^29^ In MELAS syndrome the cortex was most involved followed by multisystem degeneration and to a lesser degree the sub‐cortical structures. Multifocal infarct‐like lesions in the occipital, parietal, and temporal cortices associated with neuronal loss and gliosis was the most common neuropathological pattern in MELAS syndrome. Next to that, mineral deposits in blood vessels of the basal ganglia and capillary proliferation have been reported.^8^, ^71^ Depending on the size and distribution of the accumulation this might lead to decreased vascular lumina, increased vascular wall stiffness and disturbed perfusion.

**Table 2:** Mitochondrial angiopathy on electronmicroscopy in m.3243A>G patients

	REF
Mitochondrial accumulation in smooth muscle and endothelial cells	Ohama et al 1988^64^
Tunica media cell degeneration and necrosis	
Endothelial cell hypertrophy	Kishi et al 1988^42^
Increased number subendothelial mitochondria	
Narrowing capillary lumens	
Marked aggregation of mitochondria in smooth muscle cells and vascular endothelium	Mizukami et al. 1992^56^
Proliferation of capillary vessels	Bertrand et al. 1996^7^
Vascular sclerosis	Prayson and Wang 1988^67^

### 4.3 Neuro‐imaging in m.3243A>G migraine patients

Different types of imaging techniques have been used to study the brain of migraine patients.^50^ These include conventional imaging techniques (Computed Tomography and Magnetic Resonance Imaging/Spectroscopy and Cerebral Angiography), cerebral perfusion imaging techniques (Position Emission Tomography, Single‐Photon Emission Computed Tomography, Arterial Spin Labeling), novel imaging techniques (Oxygen Extraction Fraction) and Diffusion Tensor Imaging.^82^ In the m.3243A>G‐caused MELAS syndrome all these techniques have mainly been used in the context of stroke‐like episodes, but not in non stroke‐like‐episode associated migraine.^50^ The number of reports using brain single photon emission computed tomography (SPECT) and DTI in m.3243A>G patients without or outside stroke‐like episodes are rare.^70^

^99m^Tc‐ECD SPECT studies conducted in three patients with the m.3243A>G mutation, regardless of whether they had or had not suffered from stroke‐like episodes, showed multiple areas of asymmetrical decreased perfusion (mitochondrial vasculopathy), particularly in the posterior and lateral head regions, especially the temporal lobes. This mitochondrial vasculopathy with regional cerebral hypoperfusion may be seen on brain ^99m^Tc‐ECD SPECT regardless of whether they have or have not suffered from stroke‐like episodes.^78^ Such inappropriate intracranial hemodynamics was also observed when studying, over a period of 10 years, the natural course of five MELAS patients with hypoperfusion in the posterior cingulate cortex observed by ^99^ SPM‐SPECT.^63^ More and longitudinally repeated imaging studies using functional imaging technologies in carriers of the m.3243A>G with migraine but without stroke‐like episodes are warranted.

### 4.4 Ex vivo and in vivo brain OXPHOS (bio‐) chemistry consequences in m.3243A>G patients

Using immunohistochemistry Sparaco and coworkers showed reduced expression of COX‐II and ATPase8 in multiple brain areas of three unrelated m.3243A>G patients.^77^ The topographic analysis allowed the authors to draw some correlations between the immunohistochemical and clinical and neuropathological features, like the demonstration of a cortical mitochondrial dysfunction and the preferential involvement of the cerebral cortex in m.3243A>G patients. Betts et al.^8^ studied the neuropathology in 2 m.3243A>G patients who both died of cardiac failure. The main results were (a) a low number of COX‐deficient neurons in all brain regions, (b) no correlation between the threshold level for the 3243A>G mutation to cause complex IV deficiency within single neurons and the degree of pathology in affected brain regions. They furthermore observed that the most severe complex IV deficiency associated with the highest proportion of mutated mitochondrial DNA was present in the walls of the leptomeningeal and cortical blood vessels in all brain regions. As the most immunohistochemical studies have mainly used COX staining as OXPHOS read out general conclusions between the m.3243A>G mutation, threshold and pathology should be carefully interpreted. Indeed, it is known that due to this specific mutation also the ROS producing complexes I and III can be markedly affected. This is illustrated by Müller‐Höcker who described the results of autopsy in a nearly 13‐year‐old girl whose brain complex I activity was diminished to 20%,^60^ whereas complex IV was only slightly below the low‐normal range. It is also illustrated in a recent article of Chrysostomou et al.^18^

## 5 THE ROLE OF MITOCHONDRIAL FUNCTIONING IN MIGRAINE PATHOPHYSIOLOGY

### 5.1 Role of oxidative stress in migraine pathophysiology

Oxidative stress is implicated in the pathomechanism of several diseases affecting the brain. This organ is highly susceptible to oxidative damage, especially lipid peroxidation, because of the high content of polyunsaturated fatty acids in neurons. Elevated lipid peroxides and reduced levels of antioxidant enzymes in migraine patients have an important role of oxidative stress in the pathology of migraine (for a recent review see Ferroni et al.^28^).

Next to a direct effect on for example, lipids, increased ROS levels due to mitochondrial dysfunction can also lead to the induction of a neuroinflammatory response through the release of interleukin 1‐beta and the consequent increase in inflammatory mediators such as the prostaglandins (PG) and prostanoids.^45^ An elevated level of PGE2, the majority lipid mediator that contributes to inflammatory pain, has been reported during migraine attacks in patients suffering from migraines.^3^ Also, intravenous infusion of PGE2 was shown to cause migraine‐like symptoms in migraineurs.^2^ This highlights the role of PGE2, which might occur as a consequence of increased ROS production due to a disturbed OXPHOS, in the pathophysiology of migraine.

### 5.2 Endothelial shear and circumferential wall stress

**Figure 2:**
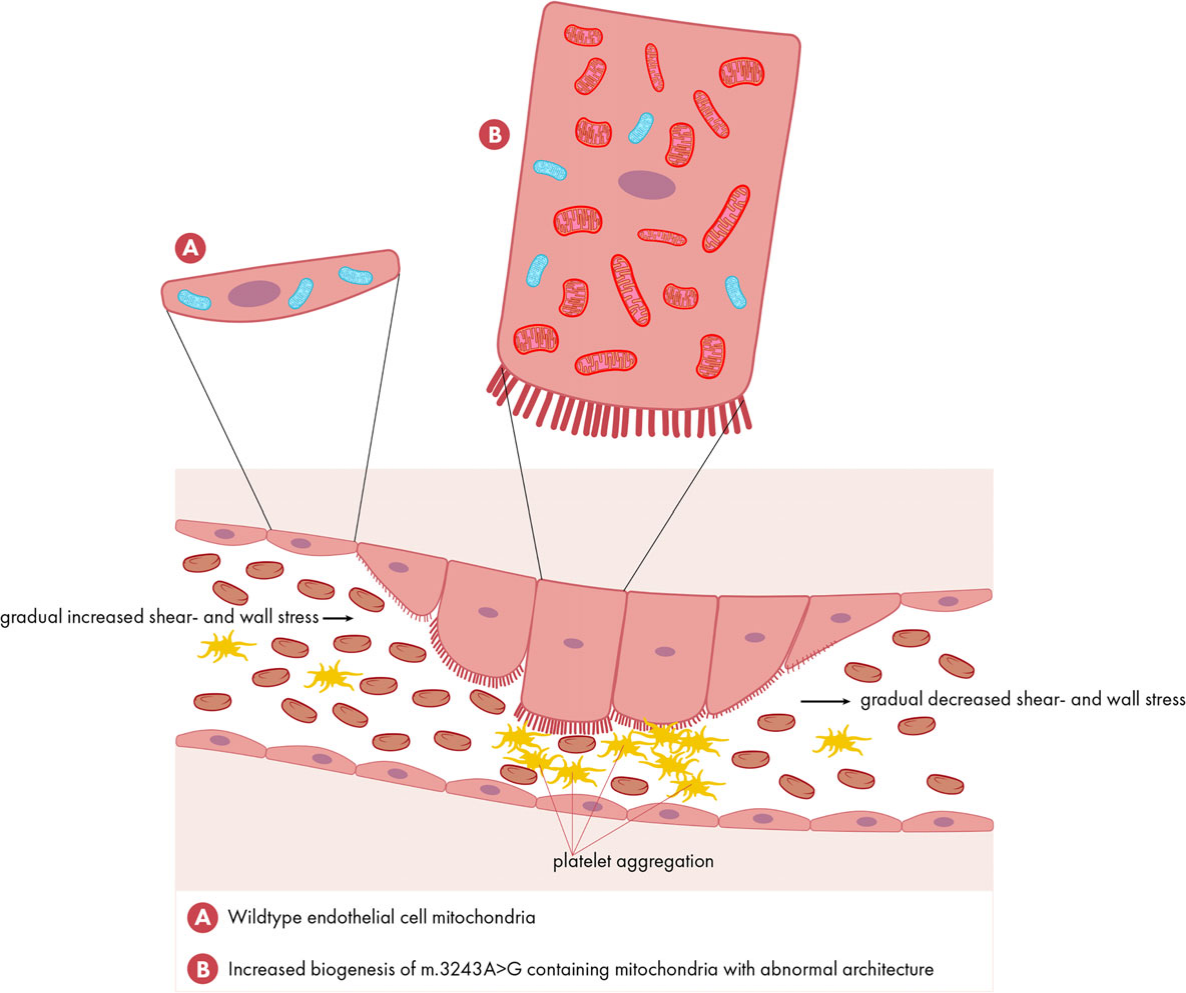
Mitochondrial biogenesis and vascular shear stress. Based on the existing heteroplasmy percentage of mitochondrial DNA molecules in m.3243A>G patients, heterotopic effects of the endothelial cells are expected. Mitochondrial biogenesis is induced by ROS. Proximal of thereby increased shear and circumferential wall stress mitochondrial biogenesis is progressively increased. Besides, the increased shear and wall stress may lead to platelet aggregation with serotonin release. Distal of the most affected endothelial cells, next to hypoxia/ischemia, decreased shear stress is expected decreasing mitochondrial homeostasis and inducing inflammation. Wild type mitochondria are depicted in blue

Blood vessels are under constant mechanical loading from blood pressure and flow that cause internal stresses (endothelial shear stress and circumferential wall stress, respectively).^48^ Wu et al.^84^ showed mitochondrial increment in cultured human umbilical vein endothelial cells subjected to laminar shear stress (LSS). The translocation of dynamin‐related protein Drp1 to the mitochondria was decreased suggesting that LSS promotes mitochondrial fusion. Besides, next to enhanced mitochondrial biogenesis they observed an increased expression of mitochondrial antioxidant enzymes and an increased mitochondrial membrane potential and ATP generation. We hypothesize that, based, on decreased perfusion in m.3243A>G patients downstream of the induced mitochondrial vasculopathy, decreased laminar shear stress causes disturbed mitochondrial homeostasis and ROS‐induced, inflammation induced impairments in brain vasculature (Figure 2). We postulate that such a disturbed mitochondrial homeostasis, leading to decreased biogenesis, expression of mitochondrial antioxidant enzymes, membrane potential and ATP production, might aggravate the preexisting mitochondrial dysfunction present in brain vasculature and neuronal cells as a consequence of the m.3243A>G mutation. Along these lines Borgdorff proposed shear‐induced platelet aggregation as one of the initiators of a sequence of events leading to a migraine attack.^10^ Shortly, increased shear stress might lead to platelet aggregation in a narrowed vessel to the brain or in the circle of Willis.^11^ The consequent release of platelet serotonin may cause pain and dilatation of the extracerebral arteries (migraine without aura) and in higher concentrations may provoke vasoconstriction. This is comparable to glutamate induced gap‐junctional slow calcium waves in astrocyte syncytium, hypothesized to be responsible for the slowly preceding aura signs.^9^

### 5.3 Cortical spreading depression

Zielman's hypothesis states that increased glutaminergic activity leads to an increased cerebral excitability and enhanced susceptibility to cortical spreading depolarization.^27^ Cortical spreading depolarization or depression (CSD) is defined as a slowly propagating (2‐5 mm/min) wave of rapid near‐complete depolarization of neurons and astrocytes followed by a period of electrical suppression of a distinct population of cortical neurons.^74^ The link between CSD and migraine aura was suggested by Milner^55^ and directly demonstrated using functional MRI in a patient during a migraine attack.^34^ Cortical spreading depression itself induces oxidative stress in the trigeminal nociceptive system (Shatillo et al., Neuroscience 253, 341‐349, 2013). Oxidative stress is also one of the consequences of hampered OXPHOS.

### 5.4 Nitric oxide, arginine and citrulline

The m.3243A>G mutation might lead to decreased NO synthesis due to impaired citrulline and arginine synthesis. Nitric oxide deficiency and consequently impaired microvasculature perfusion.^26^ To our best knowledge no randomized controlled trials have been performed evaluating the effect of arginine, citrulline, or a combination of both in the treatment of mitochondrial migraine.

## 6 MITOCHONDRIAL MIGRAINE IN M.3243A>G CARRIERS: PUTTING BITS AND PIECES TOGETHER

**Figure 3:**
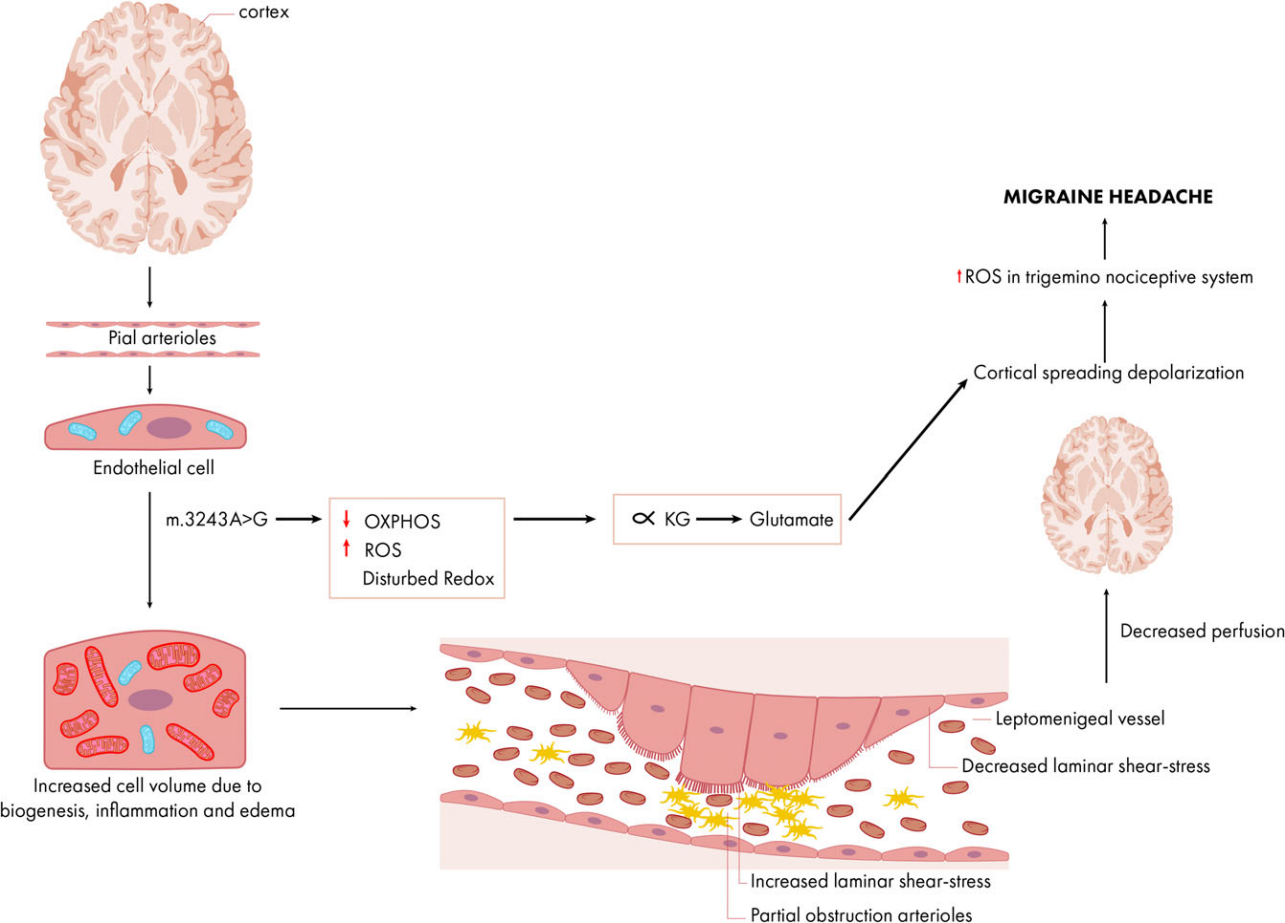
Proposed sequence of the mitochondrial disease migraine cascade. The percentages of the m.3243A > G mutation in vascular endothelial and smooth muscle cells, neurons and glia like astrocytes may vary in and between the different cell types. Long‐term exposure of toxic substances, as ROS, might contribute to the occurrence of migraine at later age. The mutation, when having passed the disease‐elucidating threshold, will lead to a hampered OXPHOS due to defective translation. A disturbance of the OXPHOS complexes, mainly complex I and III, will give rise to an increase in ROS and disturbed cellular redox‐state which cannot be compensated by the natural existent scavenger system like the superoxide dismutases. Presumably as an adaptive consequence to the altered mitochondrial architecture caused by increased lipid peroxidation, its decreased ATP generating capacity and other cell biological consequences, the affected cells will initially respond via increased mitochondrial biogenesis. In brain vascular endothelial and smooth cells narrowing of the lumen will take place (for further details see Figure 2). Next to the increased production of ROS the consequent hypoxia/ischemia, altered glutamate metabolism and ionic homeostasis might trigger cortical spreading depolarization (CSD). CSD is an activator of the trigeminovascular system leading to migraine pain^27^

Two possible pathophysiological mechanisms for migraine in mitochondrial disease have been proposed: mitochondrial angiopathy and mitochondrial cytopathy. Both conditions, if caused by an oxidative phosphorylation defect, will affect different cell types like endothelial and smooth muscle cells, neurons, and glia cells, and are, *in strictu* sensu, both cytopathies. As mitochondria play a key role in both the vascular endothelial cells, neurons and glia cells, and smooth muscle cells, the exact sequence of events finally causing migraine will be difficult to decipher. Most probably the pathological cellular consequences of mitochondrial dysfunction will act in parallel, interact with each other, be dependent on the involved cell type, might follow a different time‐scale, and will react differently on their environment. Based on available literature, with observations in humans, the following cascade of events might (partially) explain the mitochondrial m.3243A>G migraine association (Figure 3).

The first branch of our simplified migraine paradigm starts with vascular smooth muscle and endothelial cortex cell angiopathy caused by the tRNA^Leu^ mutation. Among the cell biological consequences of disturbed OXPHOS belong increased ROS and disturbed redox metabolism. Increased ROS induces endothelial cell mitochondrial biogenesis and chemical inflammation described as mitochondrial angiopathy.

Both the increased mitochondrial biogenesis and ROS‐induced inflammation and edema cause increased endothelial cell volume leading to a focal constriction (narrowing) of the lumen of the pial arterioles and small intracerebral arteries (up to 250 μm). The degree of heteroplasmy in the vascular endothelial cells presumably determines the magnitude of the narrowing due to its direct relationship with disturbed OXPHOS. As the narrowing of the vessels will lead to the subsequent effects in the proximal and distal vessel, the level of heteroplasmy may be a determinant of migraine susceptibility.

The expected increased vascular pressure proximal of the vascular narrowing causes enhanced laminar shear stress which might further progress mitochondrial biogenesis and vascular narrowing. Shear stress itself might lead to cortical spreading depolarization of neurons and astrocytes, a further increase of ROS, activation of the trigeminal nociceptive system and finally migraine. Important other mitochondrial‐dysfunction related catalysts include the increased glutamate in the visual cortex as a consequence of the hampered oxidative phosphorylation and the activation of nociceptive signaling via redox‐sensitive ion channels. Increased shear stress might also lead to platelet aggregation and serotonin release in a narrowed vessel to the brain or in the circle of Willis.^11^ The serotonin release may cause pain and dilatation of the extracerebral arteries (migraine without aura) and in higher concentrations may provoke vasoconstriction and thus being responsible for the slowly preceding aura signs.^9^

Distal of the increased endothelial cell volume a decreased shear stress is expected which might lead to decreased mitochondrial homeostasis and induces inflammation (Figure 3).

## 7 NEW ANCHORS FOR MITOCHONDRIAL MIGRAINE TREATMENTS AND TRIAL DESIGN

The first Pubmed report on treatment of migraine dates from 1888.^35^ Since then various new treatment modalities have become available for which we refer to recent reviews and guidelines.^16^, ^65^, ^75^ Based on the disentangling of the angiopathy paradigm, ROS production sites, ROS‐redox metabolism, or ROS‐induced inflammation pathways might become new targets for innovative classes of drugs to treat mitochondrial migraine in m.3243A>G patients. Such new drugs might either act alone or might be used as adjuvant to current treatment modalities.

## 8 FUTURE PERSPECTIVES

The exact sequence of events and the relative importance of factors underlying migraine in m.3243A>G patients are still enigmatic. However, substantial evidence exists in man that dysfunctional mitochondria in both the vascular, the smooth muscle cells as the neuronal system and the interaction between these are presumably at the starting point of the cascade that leads to migraine. Of the many consequences of OXPHOS dysfunction, reactive oxygen species overproduction and the thereby caused chemical neuroinflammation might become new targets for ameliorating migraine in m.3243A>G patients. Whether a similar cascade of event is applicable to other mitochondrial diseases associated with migraine warrants further studies.

## COMPLIANCE WITH ETHICS GUIDELINES

Saskia Koene, Christiaan Saris, Doug Turnbull and Mirian Janssen declare that they have no conflict of interest.

Julien Beyrath is COO and Jan Smeitink is founding CEO of Khondrion BV a mitochondrial medicine company among others developing therapies for MELAS spectrum disorders.

This article does not contain any studies with human or animal subjects performed by any of the authors.

## AUTHOR CONTRIBUTIONS

JS designed and wrote the initial version of the manuscript. SK, JB, CS, DT, and MJ each contributed to correcting and writing parts within their specific area of expertise.
